# Integrated Optical Modulator Based on Transition between Photonic Bands

**DOI:** 10.1038/s41598-018-20097-7

**Published:** 2018-01-26

**Authors:** Alperen Govdeli, Murat Can Sarihan, Utku Karaca, Serdar Kocaman

**Affiliations:** Electrical and Electronics Engineering Department, Middle East Technical University, Ankara, Turkey

## Abstract

An area efficient novel optical modulator with low operation voltage is designed based on integrated Mach-Zehnder Interferometer with a photonic crystal slab structure as the phase shifter. Plasma dispersion effect is utilized so that photonic band-to-band transition occurs at the operating frequency leading to a high index change (Δ*n* = ~4) for π-phase shift on the modulator. This approach reduces the phase shifter length to a few micrometers (~5 µm) in a silicon on insulator platform and operating voltage required is around 1 V. Low voltage together with short optical interaction length decrease optical losses and power consumption during modulation process providing a great opportunity for size and system cost optimization.

## Introduction

The transition from the electronic systems to the photonic systems has been widening for a long time. Replacing the copper based transmission lines with fiber optic cables is the prominent example. In addition, due to the limitations of the previous interconnect systems such as cross-talk, loss, low data rate and low bandwidth, new optical technologies have been developing. With the advances in the optical interconnects, the transition has begun to take place in fiber-to-device, device-to-device and chip-to-chip platforms^1^. Such systems provide better control on signal timing while providing smaller device size and lower electromagnetic interference^2–15^. Among the optical network interconnects, various studies on silicon based optical switches and modulators have been extensively conducted^16–38^. This interest in silicon is mainly due to its low fabrication cost, and high integration capability between electronic and photonic components on CMOS technology^39–44^.

The primary purpose of an optical modulator is to modulate the optical signal by altering the characteristics of the light beam such as amplitude, phase or polarization. The control of the light beam in a waveguide can be possible with the applied electric field leading to a change in the real or imaginary parts of refractive index of the material in the corresponding waveguide, which are known as electro-refraction and electro-absorption, respectively^45^. Although the change in imaginary part of the refractive index, which is associated with the absorption, can be directly used to modulate the field intensity, the variation in the real part created by various methods is preferred for the intensity modulation considering the switching applications. The traditional methods such as Pockel effect, Kerr effect and Franz-Keldysh do not provide a high enough change in refractive index of silicon to be able to implement in telecommunication systems^46,47^. Thus, different approaches are required to use silicon as modulators or switches. Due to high thermo-optic coefficient of silicon^48^, one method is thermal modulation that includes a metallic microheater placed on the silicon waveguide to obtain thermal tuning^16,49^. However, in order to eliminate the light absorption loss coming from the metallic surface, a silicon dioxide layer is placed between the heater and the waveguide, which affects the heat transfer due to the low thermal conductivity of silicon dioxide^49,50^. Therefore, this approach requires high power and also has a slow response time^45^. Other method for using silicon based devices for modulation is to utilize the plasma dispersion effect, which is the change of refractive index with the free carrier (electrons and holes) concentration^46,47^. This process could be performed with injection^19,33^, depletion^18,20–25,34,36,37^ or accumulation^17,26,27,30,32^ of the carriers.

There are mainly two popular approaches available in the literature for integrated intensity modulation. The first one is using a Mach-Zehnder interferometer (MZI), which is based on the interference of the light waves constructively or destructively for switching the output intensity. The required relative phase shift between the two light waves propagating inside the arms of a MZI is only possible with a change in refractive index. Recently, a large number of studies on MZI based modulators have been performed with phase shifter structures composed of traditional pn, p-i-n diodes or MOS structures and as a consequence, high operation speeds up to 50 Gb/s were obtained^17–27^. However, among all these designs, the shortest phase shifter length seems to be 200 µm^17,20,21^ and the lowest voltage swing for a proper modulation is 1.75V_pp_ with −0.9 V DC offset^17,20^. Furthermore, in order to increase the relative phase shift (Δ*ϕ*) between arms of MZI and reduce the length of phase shifter, in some designs push-pull drive has been employed^17^. In other words, a differential signal is applied to the arms of MZI. Although this might increase Δ*ϕ*, which results in shorter phase shifter for the same driving voltage, the average energy consumption per bit doubles.

As a part of the optimization of the MZI based modulators, photonic crystal (PhC) based structures have also been used as a phase shifter in the arms of MZI. Photonic crystals are all-dielectric structures composed of materials with different optical properties such as periodically arranged refractive index along the optic axis^51^. Among the various interesting optical behaviors that PhCs have provided, development of negative refraction is one example for such phenomena^52^. Negative refraction offers unusual physical properties such as super-focusing^53,54^, inverse Doppler effect^55,56^ and inverse Snell’s law^55^. Zero effective index either with by compensating positive phase accumulation or by utilizing Dirac cones have been also shown^57–59^. Slow light, which is associated with lowering the group velocity of the light, is offered by PhCs as well^15^. Therefore, PhCs are quite useful in terms of manipulating refractive index of the materials and there are several PhC based optical modulator designs available in the literature for enhanced performance^17,20,21,28,60–63^. For instance, length of the phase shifter was reduced to 80 µm for 1 Gb/s operation speed with 2 V on-state voltage^28^.

Beside their advantages such as high speed, wide working spectrum and low temperature sensitivity, MZI-based modulators have several major drawbacks such as their demand for long interaction length, which results in high insertion loss and occupation of large area and large power consumption. In order to eliminate such disadvantages, another approach for intensity modulation has been developed. This second approach is using a resonant structure working as a modulator with different resonant conditions at different refractive indices. Most popular resonant structures for modulation purposes are ring resonators^29–38^. Transmission of light beam in such resonators is sensitive to circumference of the ring and the wavelength of the light. If the circumference is an integer number of the propagating light wavelength, the transmission is highly reduced. These resonators are coupled to a single waveguide to modulate the optic signal^29^. Thus, after applying electric field and changing the refractive index of the ring region, one can modify the resonance wavelength. Unlike MZI-based modulators, rings have strong light confinement that results in lower power consumption and smaller chip area. In consequence, high operation speeds (50 Gb/s) with resonator based modulators have been reported where the radius of the rings are around 5 µm^29–38^. For these modulators, the voltage swing required for high speed modulation reported is usually around 2V_PP_^30,37^. On the other hand, operational wavelength range of ring resonators are quite narrow (~0.1 nm) leading to a high sensitivity to the temperature and fabrication processes^37^. Furthermore, such a narrow linewidth demand is also a critical specification in terms of the system cost.

As a result, both MZI and ring-based modulators possess advantages and disadvantages regarding the various performance specifications and this study aims to provide the advantages of both types of modulators in one structure. Proposed design includes a MZI with photonic crystal phase shifter in a p-i-n diode structure. Unlike the previous studies on optical modulators, a novel method that produces the required effective refractive index change from the band structure shift of the photonic crystal is utilized for the first time. Hence, the phase shift along a small interaction length is generated with the ability of obtaining both positive and negative refractive index in PhCs. Consequently, smaller interaction length than that of resonator based modulators and lower voltage operation than previously reported MZI based modulators have become possible.

## Negative Refractive Index and Phase Accumulation

**Figure 1:**
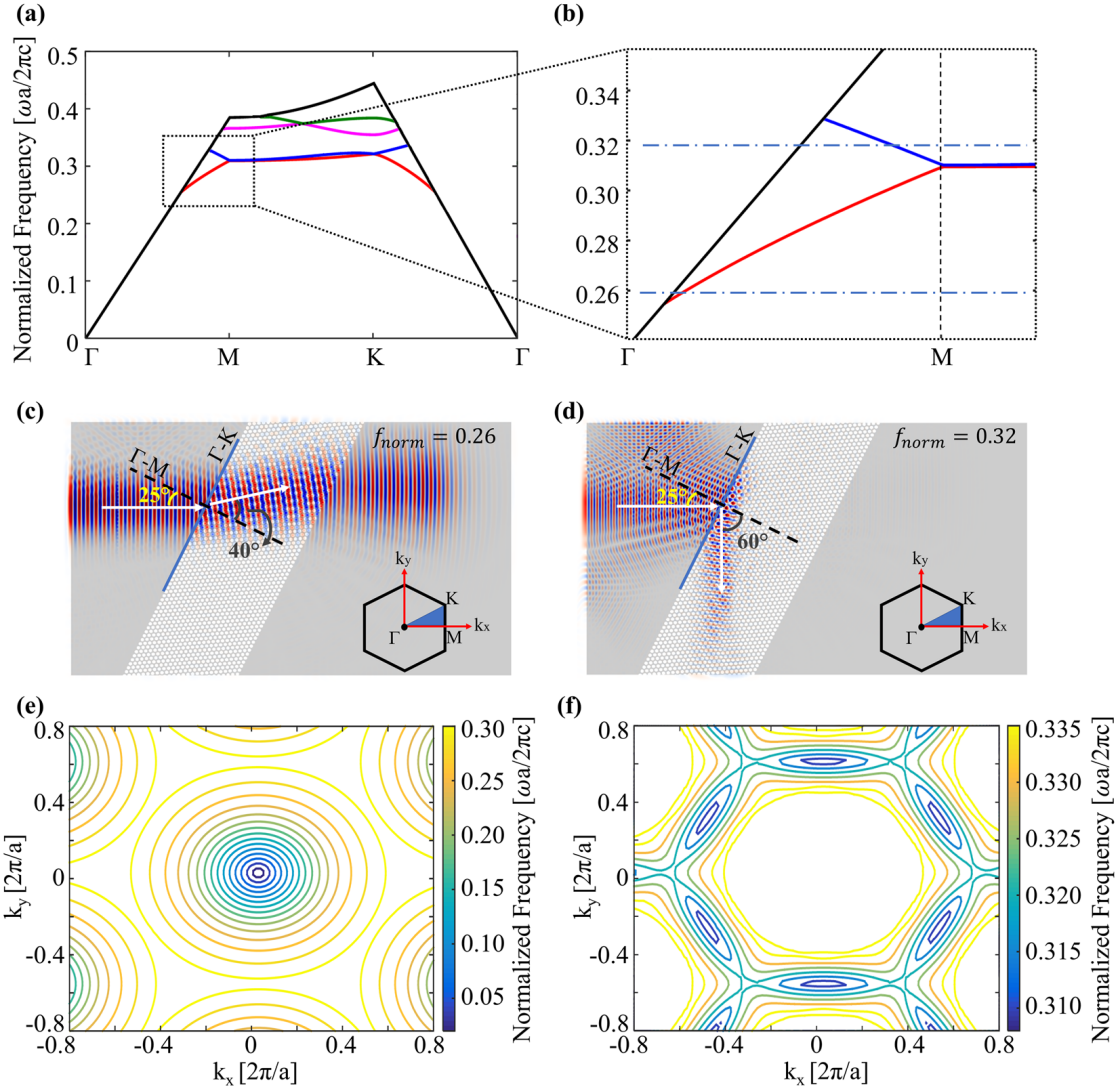
(**a**) Photonic band structure of an air-hole hexagonal lattice with *r* = 0.3a and h = 0.6a, (a = 0.5 µm) for TM-like polarization. (**b**) Zoomed view of band diagram of the region shown with dashed rectangle at (**a**). Horizontal dashed lines illustrate the normalized frequencies of 0.26 and 0.32 (*ω*a/2πc) in the first and second photonic bands, respectively. (**c**) Light propagation (field distribution) in the PhC obtained with FDTD method. The incident angle with respect to normal (Γ-M) of the interface (Γ-K) is 25° and the refraction angle is 40°. The normalized frequency of propagating light is 0.26 (*ω*a/2πc). Inset: 1^st^ Brillouin zone. The blue colored area represents one of irreducible Brillouin zones. (**d**) The same as (**c**) except that frequency of the incident light is 0.32 (*ω*a/2πc) and the refraction angle is −60°. Light is refracted through the same side of the normal line (Γ-M) as the incident light comes (negative refraction). (**e**) The EFC plot of TM-like modes in the same PhC at frequency range of 0.05–0.30 (*ω*a/2πc) – the first photonic band. (**f**) The EFC plot of TM-like modes at frequency range of 0.310–0.335 (*ω*a/2πc) – the second photonic band.

Consider a PhC structure with 2D hexagonal lattice of air-holes etched into a silicon-on-insulator (SOI) platform. The radius of holes is *r* = 0.3*a* and the thickness of silicon slab is *h* = 0.6a where the lattice constant is a = 0.5 µm (normalized frequency of 0.322 (*ω*a/2πc) corresponds to 1550 nm wavelength). The photonic band diagram on which the behavior of light (for TM-like polarization) inside the PhC directly depends is shown in Fig. 1a,b. Refraction of the incoming light into the PhC structure can be determined by the slope of the band associated with the frequency of the light^64^. Thus, at Γ-M direction, the effective refractive index of PhC is expected to be positive and negative sign for the first and second photonic bands, respectively^64^ (see Supplementary Information). In order to visualize the polarity of refraction, one frequency at each band was chosen (0.26 and 0.32 (*ω*a/2πc) for positive and negative refractions, respectively) and the propagations of TM polarized electric field were calculated with finite-difference time-domain (FDTD) method. In these simulations, the PhC structures were exposed to a light source in a direction such that incidence angle is 25° and the results are shown in Fig. 1c,d. Propagation in Fig. 1c is for the positive refraction and optical path is similar with the ray optics analysis of any natural material. However, unlike in Fig. 1c, as one can observe from the Fig. 1d, light enters the PhC and propagates along the direction in the reverse side of the normal line (Γ-M), which is the indication of negative refraction. After applying Snell’s law with incidence and refraction angles shown on Fig. 1c,d, the effective refractive indices are calculated as 1.41 and −1.25 at 0.26 and 0.32 (*ω*a/2πc), respectively.

Next, equi-frequency contours (EFCs)^65,66^, the other important tool to analyze PhCs, were also utilized and the results for the PhC composed of air holes etched on SOI to form a hexagonal lattice with *r* = 0.3*a* and *h* = 0.6a are shown in Fig. 1e,f. Both sign and magnitude of effective index can be obtained from the contours. Since radii of contours decreases as frequency increases for the frequencies at the second photonic band, the PhC has a negative effective refractive index (Fig. 1f)^65^. On the other hand, contours have higher radius values for the higher frequencies at the first band meaning that PhC provides positive refraction (Fig. 1e) (see Supplementary Information). According to EFCs, the PhC possesses effective indices of 1.61 at 0.26 (*ω*a/2πc) and −1.63 at 0.32 (*ω*a/2πc). Moreover, shape of contours gives the information about the dependence on incidence angle as well^65,66^. As all the contours are circles in the first band, same index value is expected for different incidence angles in the first band (Fig. 1e) whereas refractive index varies with incidence angle more in the second band due to the nonuniform shape of the contours (Fig. 1f). This is probably why the index difference between values calculated via Snell’s law (Fig. 1d) and found from EFCs (Fig. 1f) for the second band are relatively higher. Nevertheless, the results from two different methods both demonstrate the negative refraction and calculated values are considerably close to each other.

During the propagation of the light, a phase accumulation occurs depending on the refractive index of the medium as given in the Eq. 1.

1 $${\phi }={n}_{eff}\frac{2\pi }{\lambda }d$$

where *φ* is the accumulated phase for the distance of *d* along the material. Like field distributions in Fig. 1c,d, the effect of refractive index on the light can also be observed when light is propagated along the optic axis (Γ-M) and the effective refractive index can be determined with Eq. 1 (see Supplementary Information).

**Figure 2:**
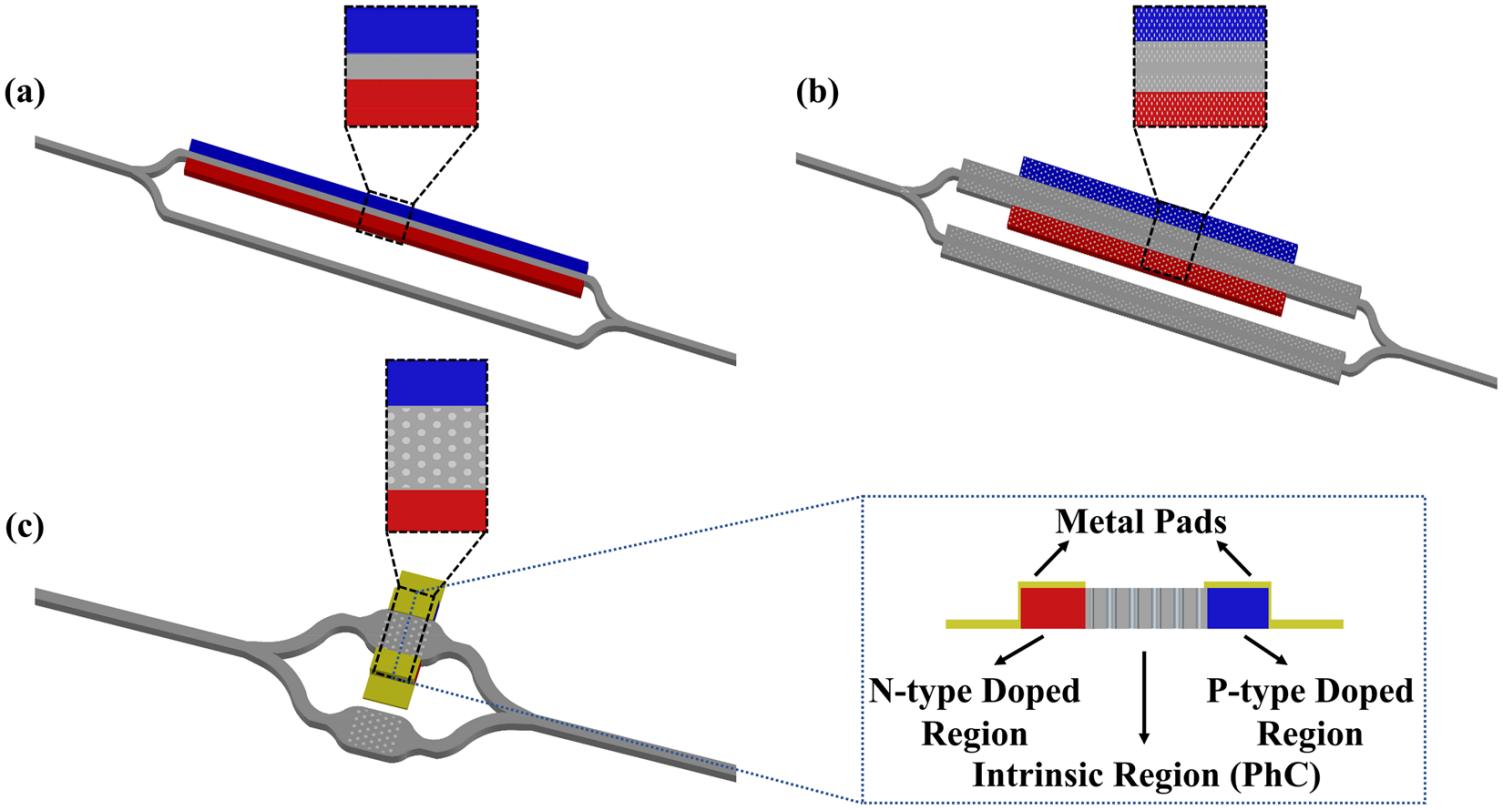
Various MZI based optical modulator structures. Blue (dark) and red (light) regions represent the p-type and n-type doped regions, respectively. (**a**) Simple Mach-Zehnder Interferometer (MZI) including silicon waveguide as a phase shifter in a p-i-n diode structure. With the silicon index change of 0.01, required minimum interaction length is approximately 78 µm for a proper modulation process (i.e. creating a π phase difference between two arms of MZI via electric signal). (**b**) MZI with line-defect photonic crystal waveguides on both arms. Phase shift is created via plasma dispersion effect with electric signal applied to p-i-n diode structure on one arm. The phase shifter length is 80 µm^28^. (**c**) The schematic representation of the proposed design which consists of a MZI with PhC on its arms. Phase shifter length is ~5 µm. Inset: p-i-n diode structure for free carrier accumulation for PhC slab waveguide. Plots are not drawn to scale.

Band diagrams, Snell’s Law-based calculations on field distributions and EFCs show that for frequencies in different photonic bands of the PhC, the effective refractive index vary substantially and this huge index change can be useful to provide distinctively different phase accumulations on an optical system. Furthermore, the phase accumulation on the PhC can be controllable via applied electrical signal and such a phase difference can be utilized for the on and off conditions of a Mach-Zehnder Interferometer (MZI) based intensity modulator. For the devices in silicon photonics, since the refractive index variation of bulk silicon is low with alternating electric field, as mentioned above, change in the carrier concentrations is the effective way of controlling the refractive index (plasma dispersion effect)^46^. According to the experimental results of Soraf and Bennett^46^, the accumulation of free electron and hole carriers with densities of 4.1 × 10^18^ cm^−3^ creates refractive index change of 0.01 in silicon at wavelength of 1550 nm. Such a change in index requires a long waveguide (minimum ~78 *μm*–Fig. 2a) for π phase shift. Thus, a MZI structure consisting of Si waveguides will take a considerable space on the chip and is not ideal as a modulator. Furthermore, optical modulators composed of MZI with PhC phase shifter so far have been usually designed to make use of the slow light effect^17,20,21,28,60–63^. Among various studies on slow light with PhC, the shortest phase shifter seems to be 80 µm-long phase shifter composed of line defect photonic crystal waveguide for 1 Gb/s operation speed with 2 V on-state voltage (Fig. 2b)^28^.

## Results and Discussion

### Required phase change

**Figure 3:**
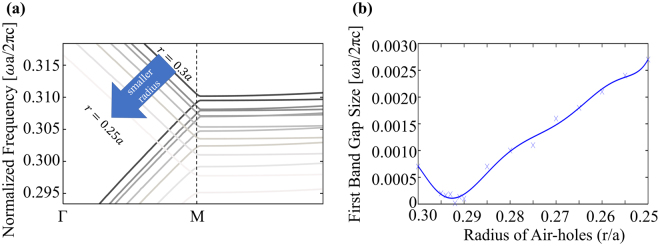
(**a**) Band structures (first two photonic bands) of PhCs with thickness of 0.6a on SOI for various air-hole radius values. Band diagrams shift below for descending radius. (**b**) Band gap size behavior for different air-hole radius. The minimum gap occurs when the radius is 0.292a.

Here, we propose a design such that two arms of a MZI are composed of identical PhCs. In order to use a PhC as a phase shifter, PhC on one arm is sandwiched between p-type and n-type doped region. Therefore, the structure is simply a p-i-n diode (Fig. 2c) that works at the forward bias mode to increase the free carrier concentration in the intrinsic region (PhC). Schematic representation of the proposed design is shown in Fig. 2c. Unlike previous designs, this design is based on the photonic band to band transition for the optical modulation. In other words, the change in silicon index is utilized to shift the photonic band structure of the PhC and the transition from one band to another band is obtained at the operating frequency. As a result, higher effective refractive index change in PhC slab is possible since both magnitude and sign of the effective index change considerably between first and second photonic bands as mentioned in the previous section. Although the majority of the studies on the photonic crystals focus on maximizing the band gap^65^, the aim here is to minimize the band gap as the operating frequency will switch bands during modulation. Hence, starting from the PhC given in the previous section, radius of the air-holes was optimized and Fig. 3a summarizes the behavior of the band structures for various radii. The plot for the bandgap width between first and second photonic bands for the cases in Fig. 3a is shown in Fig. 3b. As a result, ideal radius for PhC to be used for band-to-band transition based phase shifter seems to be 0.292a where the lattice constant can be determined as *a* = 0.4774 *μm* for modulation of optical signal carried at 1550 nm (normalized frequency of 0.3080 (*ω*a/2πc)).

**Figure 4:**
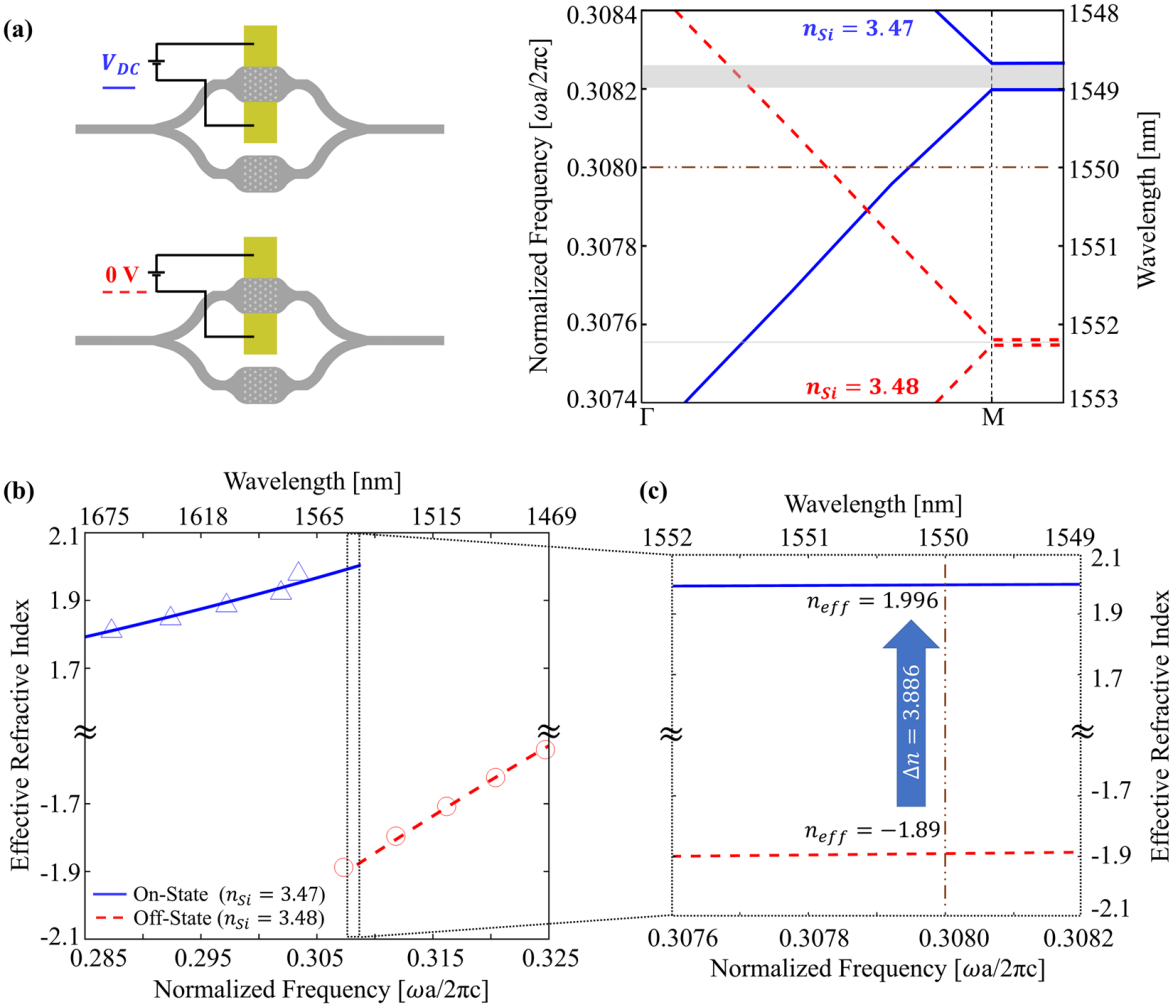
(**a**) Schematic representations of MZI-based proposed modulator and the band structure of the phase shifter in the MZI at different operation states. Phase shifter is a hexagonal air-hole slab lattice with *r* = 0.292*a*, *h* = 0.600*a* where *a* = 0.4774 *μm*, for TM-like polarization. Silicon index is 3.48 at off-state of the modulator (red-dashes). Index of the silicon becomes 3.47 at on-state (blue-solid) with the applied voltage *V*_*DC*_. Shaded areas in the plot of band diagram represent bandgaps in the band structures. (**b**) Effective refractive index of the phase shifter PhC at off-state (red – circles, dashes) and on-state (blue – triangles, solid) of the proposed modulator. Red and blue lines represent the fitted data based on the effective indices. (**c**) Focused view of the area covered with dashed rectangular in (**b**). It is the region that phase shifter PhC has positive and negative effective index depending on state of the modulator. In other words, it is the frequency interval which is in the second and the first photonic bands of off-states and on-states of the modulator, respectively (active region). Effective index is shifted from −1.89 to 1.996 at 1550 nm with the applied voltage, which means an index change of 3.886 between two states.

Based on the results of Soraf and Bennett^46^, refractive index of the device (silicon) layer alters from 3.48 to 3.47 if the applied voltage provides 4.1 × 10^18^ cm^−3^ free carriers. Such change in silicon index shifts the band diagram of the PhC used as phase shifter in MZI such that PhC working at the second photonic band with no bias voltage (Fig. 4a-red, dashes) starts working at the first band (Fig. 4a-blue, solid) with the applied voltage. This transition creates a large index change (${\rm{\Delta }}{n}_{eff}= \sim 4$) between two states at 1550 nm, although change in silicon index was only 0.01 (Fig. 4c). Effective index of PhC and its change is shown in Fig. 4b,c. This amount of shift in effective index enables us to reduce the interaction length substantially compared to other MZI based modulators (Fig. 2a,b). For π radian-phase shift during modulation, less than 200 nm phase shifter length is theoretically enough with such high index change. However, the length of the structure designed here need to be several micrometers to facilitate an effective photonic crystal functionality. Therefore, the PhC designed here has the length of 5.788 µm (7*a*√3), width of 1.91 µm (4*a*), and thickness of 0.286 µm (0.6*a*). The transition from single-mode waveguide to the PhC can be performed adiabatically to reduce the loss during transition^57^. An important note is that the change in the magnitude of the effective indices is at least one order of magnitude higher than the change in the bulk silicon.

### The p-i-n diode and DC operation specifications

**Figure 5:**
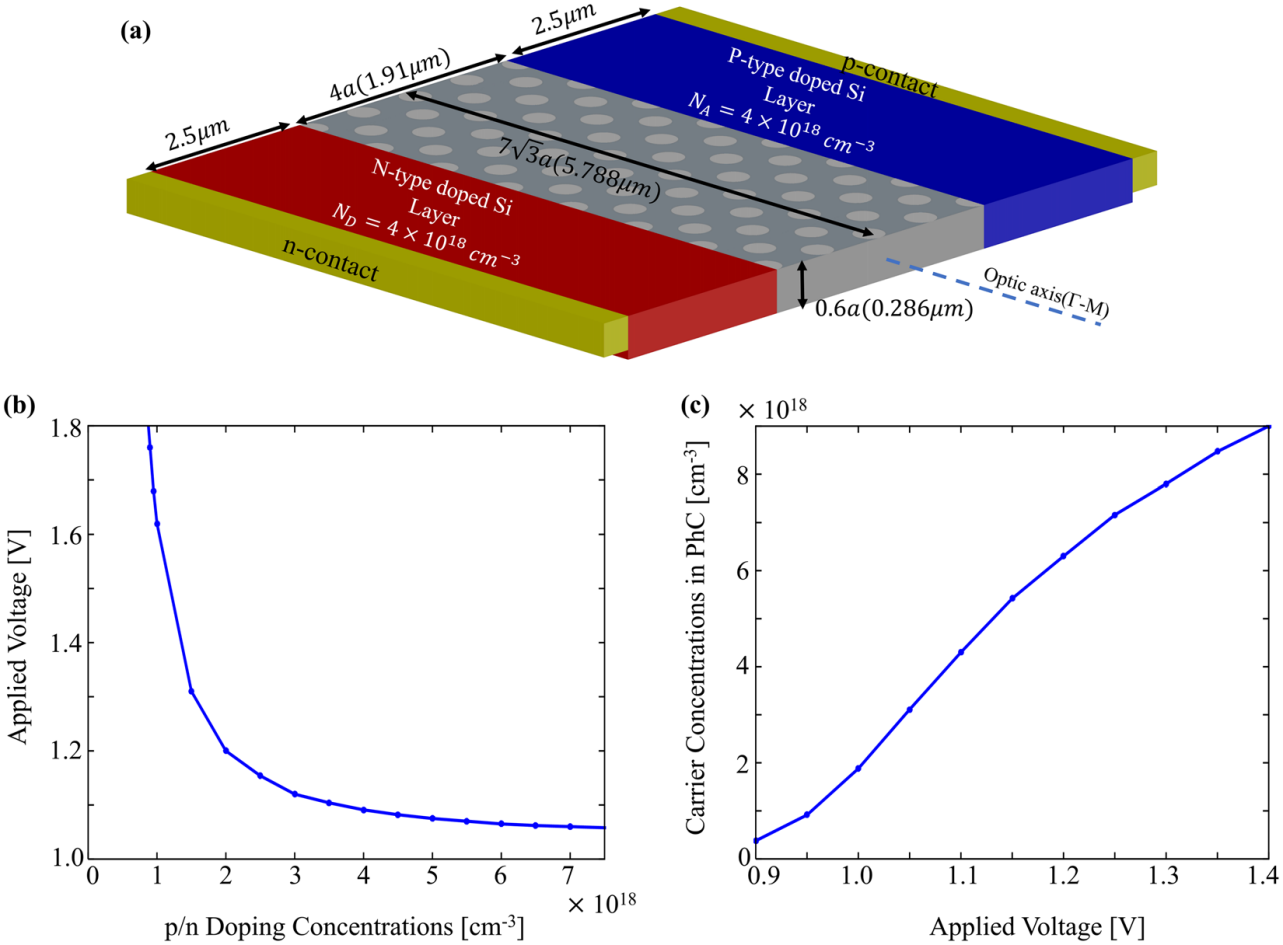
(**a**) p-i-n diode structure schematic representation with doping concentrations and sizes. (**b**) Relation between the doping density of doped regions and applied voltage required to accumulate free carrier density of 4.1 × 10^18^ cm^−3^ in the intrinsic region. (**c**) Behavior of carrier concentration in the intrinsic region for various applied voltage to the contacts.

The light intensity modulation with the proposed design is planned to be performed with the help of a p-i-n diode structure as previous designs in the literature. The intrinsic region of the diode designed here is composed of a PhC slab and the aim of the p-i-n diode (Fig. 5a) is to introduce free carrier with density of 4.1 × 10^18^ cm^−3^, which results in a silicon index change of 0.01, as uniform as possible to the intrinsic region. For this process, the diode should be operated under forward bias which is going to improve the diffusion of the majority carriers^32^. Therefore, the positive voltage is applied to p-contact while n-contact behaves as negative terminal. The relationship between p-n doping densities and bias voltage providing free carrier concentration of 4.1 × 10^18^ cm^−3^ is shown in Fig. 5b (see Supplementary Information). As expected, the higher the doping densities of p and n regions are, the lower required voltage value is. On the other hand, if the doping densities in the regions increase, the uniformity of the carrier distribution has been observed to be deteriorated. In other words, there exists a trade-off between minimum voltage required for modulation and uniformity of the carrier distribution in the intrinsic region. After running numerical simulations with a variety of different parameter pairs, doping concentrations for both p-type and n-type doped regions were determined as 4.1 × 10^18^ cm^−3^ for the lowest switching voltage with flat carrier distribution within the intrinsic (PhC) region of the p-i-n diode. At this doping density, the carrier concentration injected to intrinsic region (PhC) for different voltage values is given in Fig. 5c. Carrier density of 4.1 × 10^18^ can be obtained with 1.091 V, which is lower than operation voltages of optical modulators reported in the literature so far.

### Overall design

**Figure 6:**
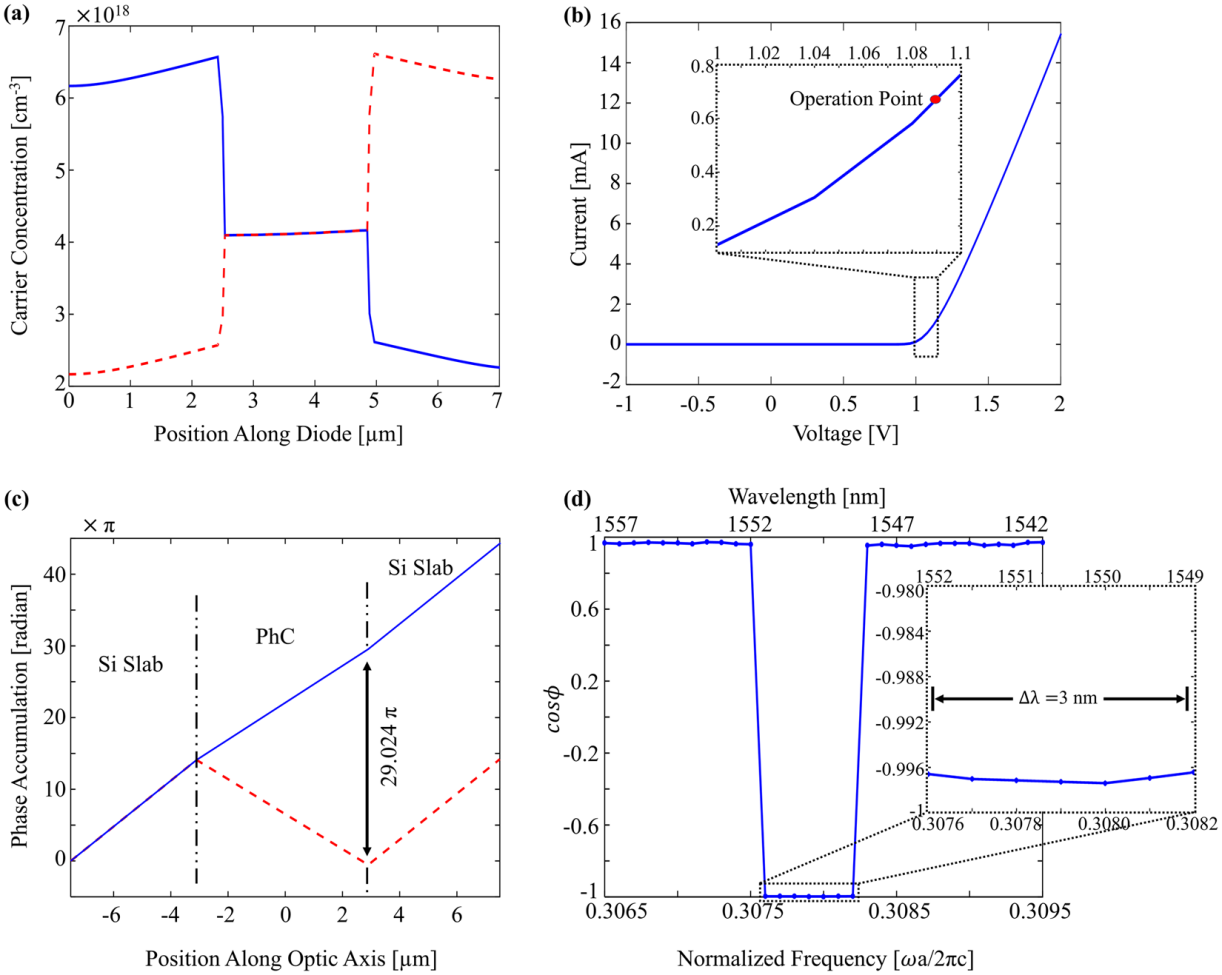
(**a**) Hole (red-dash) and electron (blue-solid) carrier distribution in the diode along the direction (Γ-K) perpendicular to optic axis. (**b**) Current-Voltage characteristics of the diode. The operation point is represented with red dot (1.091 V, 0.66 mA). Inset: I-V characteristics focused to the DC operation point. (**c**) Phase accumulation at the arms of the MZI along the optic axis during on-state at 1550 nm. (**d**) Phase difference between waves in two arms of the proposed MZI at on-state for the 1542–1557 nm range. *cosϕ* takes values between −0.9964 and −0.9974, which are associated with out-of-phase state. The active region for modulation is 1549–1552 nm interval.

The specified parameters for the p-i-n diode provided the required carrier concentration (4.1 × 10^18^ cm^−3^) uniformly along the intrinsic region of the diode (PhC) at the on-state of the modulator (Fig. 6a). The current-voltage characteristics in Fig. 6b show that 0.66 mA current passes through p-i-n diode for the required phase difference. The forward bias resistance of the diode can be determined as 125 Ω again from the Fig. 6b. Under the dc operation conditions, such resistance corresponds to a DC power consumption of 54 µW. The diffusion capacitance of the corresponding diode due to the carriers accumulated in the intrinsic region was calculated as 24 pF at 1.091 V^67^. During the switching from 0 to 1.091 V, an additional power is consumed. For such a capacitance, this power is extremely lower than the DC power consumption. The optical insertion loss along the MZI is examined for both operation states of the modulator via FDTD simulations. Output field intensity has been compared with the source and the loss at the PhC regions has been calculated to be ~1.08 dB (~1.05 dB) for the OFF (ON) state including the reflections in the interfaces. Therefore, even though a photonic band gap material is utilized in the modulator, as the operating frequency always stays in the high transmission region of the band diagram, the optical transmission is not affected.

Once the dc operation parameters are determined for the proposed structure, the optical dynamics for a proper modulation on the proposed MZI-based modulator can also be calculated. The phase difference of waves in two arms of the MZI and the output intensity has the following relation.

2 $${I}_{1}+{I}_{2}+2\sqrt{{I}_{1}{I}_{2}}cos\varphi $$

where *I*_1_, *I*_2_ and *ϕ* are the intensity of the waves propagating along interferometer arms and phase difference between the waves (${\rm{\Delta }}\varphi =\frac{2\pi }{\lambda }{\rm{\Delta }}nL$), respectively. At the off-state (no-voltage) waves are in-phase (maximum intensity). The applied voltage changes the state of the modulator (on-state) for the 5.788 µm (7*a*√3)-length PhC and at the end of the PhCs, there exists almost exactly 29π radian difference between phases accumulated in two arms of the MZI (Fig. 6c). In other words, waves become out-of-phase (*cosϕ* = −0.9974), which means minimum output intensity, at 1550 nm. Figure 6d shows the spectral phase difference for the proposed structure. The intensity contrast between off-state and on-state is high enough to modulate optic signal for an operation bandwidth (Δ*λ*) of 3 nm, which is quite larger than a typical ring-resonator based optic modulator provides.

Switching time between on and off states is clearly one of the most critical features of a modulator designed for optical communications. Since the proposed modulator here relies on the carrier accumulation, the modulation rate can be determined by the p-i-n diode characteristics. Time dependent simulations (see Methods) show that time required to switch one state to another has been calculated as 2 picoseconds. In other words, 250 GHz switching (ON and OFF) for one optical channel is possible and depending on the number of channels available in the optical bandwidth, terahertz modulation rate can be easily achieved^68^. Furthermore, the operation bandwidth can be extended as well by increasing the voltage applied for the on-state of the modulator to be able to switch more optical channels simultaneously for higher modulation speeds. For instance, by applying 1.4 V (2.4 V) instead of 1.091 V, the bandwidth can be increased from 3 nm to 5 nm (11 nm).

**Figure 7:**
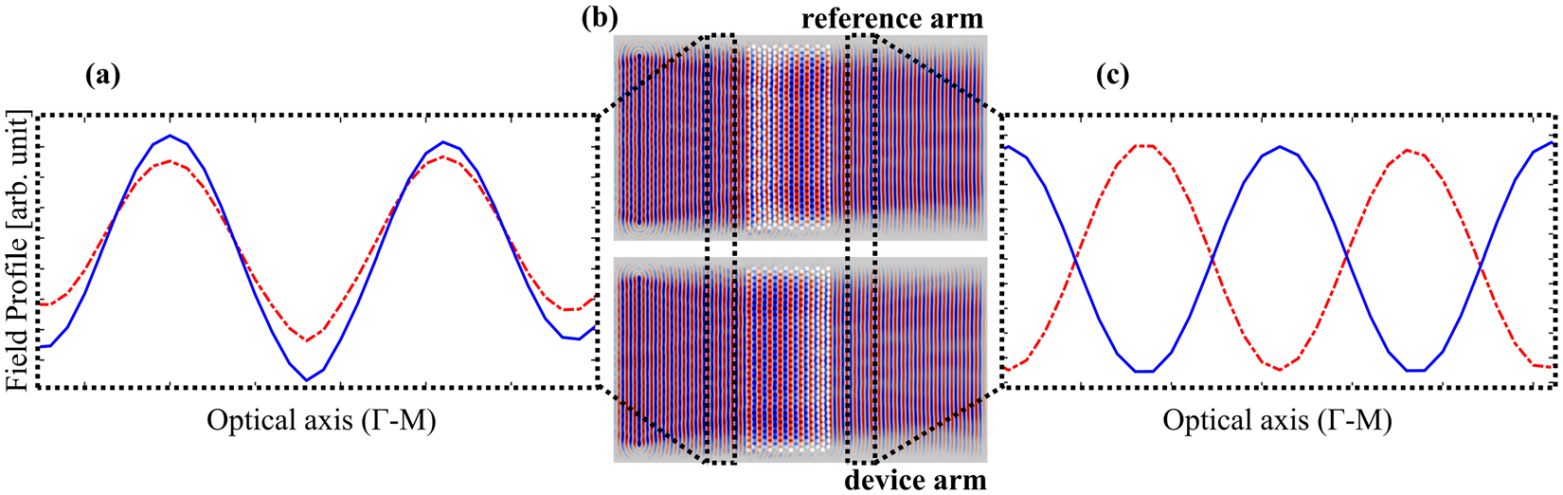
(**a**) Electric field profile of the waves in the reference (red-dash) and device (blue-solid) arms of the MZI during on-state (*V*_*DC*_ = 2.4 *V*) at the input side of the phase shifter region (PhC) along the optical axis. (**b**) Field distribution in two arms of the MZI. (**c**) Field profile in both arms at the output of the phase shifter region (PhC) along the optical axis. The operation wavelength is 1550 nm. There exists a π radian phase difference between the waves after phase modulation.

Finally, in order to demonstrate modulation of the phase further, field distributions on the device have been also examined for an operation voltage of *V*_*DC*_ = 2.4 *V* at 1550 nm and the results are summarized in Fig. 7. The field distributions of the waves propagating along the reference arm (no p-i-n diode) and device arm (with biased p-i-n diode) of the MZI clearly show that phase shifter region creates a π radian phase difference between waves in two arms of the MZI (Fig. 7c) at the ON state which is transformed into intensity contrast at the output.

In summary, band-to-band transition in a bulk photonic crystal structure is shown to be utilized as a phase shifter component in a MZI for intensity modulation. The phase shifting is based on the carrier injection process via forward-bias of a p-i-n diode structure with intrinsic region composed of a photonic crystal. The plasma dispersion effect that has been used in a large number of recent studies on optical modulators was utilized for the first time to change the band structure of the photonic crystal so that band-to-band transition could occur and interaction length for a proper modulation process could be reduced to a few micrometer lengths. Moreover, operation voltage for the modulation has been also reduced compared to the previously reported designs. Thus, the optical losses and power consumptions during modulation can be drawn lower levels supporting the efforts for realization of large-scale all-optical integrated circuits.

## Methods

### Photonic band structures

The band diagrams in Figs 1a,b and 4a were calculated using MIT- Photonics-Bands (MPB) program^69^, which is a free software that computes eigenfrequencies of Maxwell’s equations in periodic dielectric structures for k-space. The three-dimensional simulations were conducted for TM-like photonic bands. The resolution of all simulations was set as 32 grids/µm and 500 k-points were used to interpolate.

### EFCs and effective refractive index calculations inside PhCs

An EFC is a contour plot of eigen-frequencies of Maxwell’s equations in wave vector space, namely k-spice. Unlike band diagram, it includes all k-points covering the specified k-spice, not just irreducible Brillouin zone.

Here EFCs were obtained via MPB with the resolution of 32 grids per micrometer. The contours for frequencies outside of the light cone were excluded. K-space was determined in the borders of ±1 (2π/a) for both *k*_*x*_ and *k*_*y*_ axis. During the MPB simulations, first, each k-point inside these borders was formed and a map covering all such points was created. Then, for all photonic bands, eigen-frequencies of Maxwell’s equations were found point-by-point. The resulting 100 × 100 matrix was plotted via a plotting software. The effective refractive indices were determined from the relation ${r}_{EFC}=|k|=\omega {n}_{eff}$ where *r*_*EFC*_ is radius of corresponding contour and *k* is in the first Brillouin zone. The sign of the indices was determined from the behavior of the contours with increasing frequency. If they move outward, the sign is positive. Otherwise, the sign is negative^65,66^. (see Supplementary Information).

### Electric field distributions

The field distributions in Figs 1c,d and 7 were obtained using MEEP^70^, which is a free finite-difference time domain method (FDTD)-based software package. In all numerical simulations, a uniform computational grid of 24 grid points per micrometer were used. The simulated structure was a silicon on insulator (SOI) slab acting as an asymmetric planar waveguide. A continuous plane wave as a source was introduced to the structure, which excites TM-like modes. Perfect matching layers (PMLs) were placed along the x-directed and z-directed boundaries. In order to eliminate numerical reflections occurring during propagation of wave-fronts parallel to PML, an absorbing boundary condition was used at the y-directed boundaries.

### Change in refractive index of bulk silicon due to the plasma dispersion effect

Carrier concentration required to obtain a specific index change in silicon bulk at the wavelength of 1550 nm was determined from the relation ${\rm{\Delta }}{n}_{Si}={\rm{\Delta }}{n}_{e}+{\rm{\Delta }}{n}_{h}=-[8.8\times {10}^{-22}\times ({\rm{\Delta }}{N}_{e})+8.5\times {10}^{-18}\times {({\rm{\Delta }}{N}_{h})}^{0.8}]$ where Δ*n*_*e*_ and Δ*n*_*h*_ are silicon index changes due to electron and hole carrier concentration, respectively. Δ*N*_*e*_ and Δ*N*_*h*_ are accumulated electron and hole concentrations, respectively when a forward bias V_DC_ is applied^46^. (see Supplementary Information)

### Determination of p-i-n diode specifications

The analysis of the p-i-n diode utilized in the modulator structure was performed via Synopsys Sentaurus device simulator^71^. The Poisson and electron-hole continuity equations were solved in a coupled way in order to obtain electrostatic potential and electron/hole concentrations and to extract the device characteristics. The continuity equations [$\frac{dn}{dt}=\frac{1}{q}\nabla .(q{\mu }_{n}nE+q{D}_{n}\frac{dn}{dx})$ and $\frac{dp}{dt}=-\frac{1}{q}\nabla .(q{\mu }_{p}pE-q{D}_{p}\frac{dp}{dx})$] coupled to Poisson equation [$\nabla {\rm{\varepsilon }}\cdot \nabla \rlap{/}{0}=-q(p-n+{N}_{D}-{N}_{A})$] under applied initial bias were solved as a first step via Sentaurus device simulator, and by taking the previous solution as an initial value, the set of solutions were converged after iterative simulations. Furthermore, for the recombination process radiative, Auger and Shockley-Read-Hall (SRH) recombination mechanisms were taken into account during the simulations (see Supplementary Information).

The relation between the bias voltage between the contacts of the diode and the charge accumulated in the intrinsic region is $C=\frac{dQ}{dV}$ where *C* is the diffusion capacitance due to the carriers in the intrinsic region. The carrier density for different biasing voltage values (Fig. 5c) were obtained via Sentaurus device simulator. The behavior of the total charge in the intrinsic region was determined via the relation *Q* = q × (*n* + *h*) × V where *q*, *n*, *h* and V are electron charge (1.6 × 10^−19^ C), electron density, hole density and volume of the intrinsic region, respectively. For the proposed design, electron and hole carrier densities are equal in the intrinsic region. The slope of the total charge versus biasing voltage plot at the operation voltage (1.091 V) gives the capacitance (See Supplementary Information). The resistance of the diode was determined from the slope of the current-voltage graph (Fig. 6b) at 1.091 V. In other words, $R={({\frac{dI}{dV}|}_{V=1.091})}^{-1}$ (See Supplementary Information).

### Data Availability

All data generated and analyzed during this study are included in this published article (and its Supplementary Information files).

## Electronic supplementary material

Supplementary Information
