# Supplementary material for: Integrated Optical Modulator Based on Transition between Photonic Bands
Source: Sci Rep. 2018 Jan 26;8:1619. doi: 10.1038/s41598-018-20097-7 (PMC5786106; doi:10.1038/s41598-018-20097-7)
Supplement: Supplementary file 1 — Supplementary Information [file 41598_2018_20097_MOESM1_ESM.pdf]

# Integrated Optical Modulator Based on Transition between Photonic Bands

Alperen Govdeli[1], Murat Can Sarihan[1], Utku Karaca[1], Serdar Kocaman[1*]

[1] Electrical and Electronics Engineering Department, Middle East Technical University, Ankara, Turkey

* Corresponding author: skocaman@metu.edu.tr

# Supplementary Information

## I. Equi-frequency Contours and Effective Index Calculations

A photonic band structure of a photonic crystal (PhC) is the plot of eigen-frequencies of Maxwell's equations and provides information regarding supported optical modes and behavior of the light inside the corresponding PhC only along the boundaries of the irreducible Brillouin zone, which is defined by Γ (0,0), M (0,0.5) and K (-1/3,1/3) for triangular lattice like the lattice mentioned in the main article (See Fig. 1a-b).

An equi-frequency contour (EFC) also shows the frequency $\omega(k)$ solutions of Maxwell's equations in wave vector space (k-space). On the contrary to the band structure, an EFC includes $\omega(k)$ for the all k-points inside the chosen k-space (Fig. S1). It provides important details on optical properties of a PhC such as propagation direction of light inside PhC and phase refractive index $n_p$ [65,66].

The gradient vector of EFC at specific frequency gives the group velocity at that frequency for the light propagating inside PhC ($\vec{v_g} = \nabla_{\vec{k}}\omega$). The relation between group velocity and group index is $|v_g| = c/|n_g|$. In the article, we have focused on the phase index ($n_p$) as the effective index. Phase index can be calculated from the EFCs. The radius of each contour represents the wavenumber (eq. S1) [66].

$$r_{EFS} = |k| = \omega n_{eff} \qquad \text{(S1)}$$

The sign of the effective index is associated with the directions of group velocity and the wave vector inside the PhCs. In other words, sign ($v_g \cdot k$) function determines the sign of the effective index. The relation between the directions of $v_g$ and $k$ can be deduced from the EFCs. If the contours move outward with increasing frequency, refractive index is positive ($n_p > 0$). Otherwise, the sign of refractive index is negative ($n_p < 0$) [66]. Such difference in the behavior of the contours can be seen in frequencies at the first and second band (Fig. S1). For instance, effective index is positive at first band since $v_g$ and $k$ are in the same direction. The negative index occurs in the corresponding PhC at second band as can be seen in the EFC of the second band.

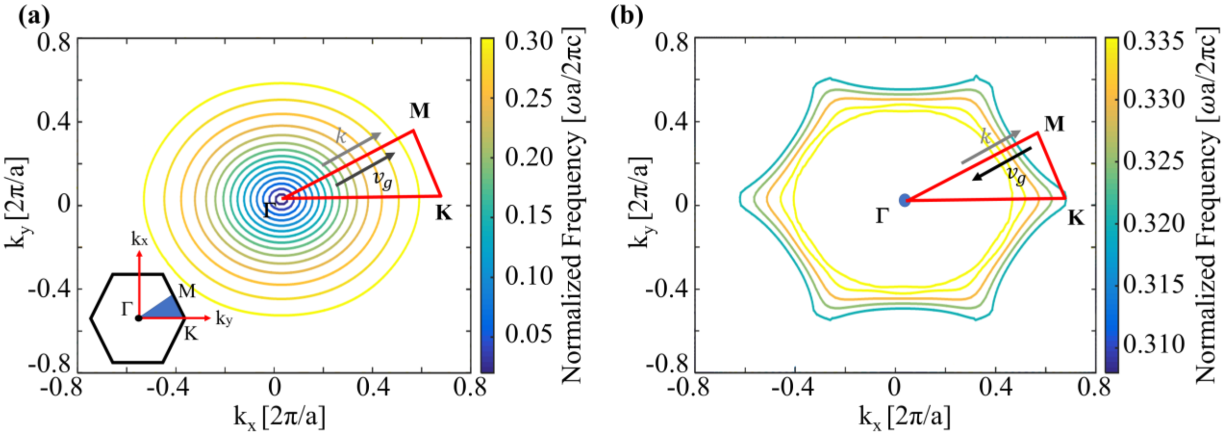


**Figure S1.** EFCs for the silicon PhC composed of hexagonal lattice of air-holes with $r = 0.3a$ and $h = 0.6a$ for the frequencies at first (a) and second (b) photonic bands (first Brillouin zone). The irreducible Brillouin zones are covered via red lines. The wave vector and group velocity directions are illustrated to distinguish the positive and negative refractive indices.

## II. Field Distribution and Phase Accumulation

The electric field distributions in the PhCs were obtained in order to ensure the refractive index and phase accumulation in the corresponding PhCs. The field distributions were determined via finite-difference time domain method (FDTD) for two different frequency. Each red and blue colors on distributions represent the peaks and deeps of the electric field propagating along the structure, respectively (Fig. S2). The locations and magnitudes of the field were extracted. The propagation of the electric field is sinusoidal. Therefore, with each consecutive peak, a 2π radian-phase is accumulated. The relation between phase difference and the length of corresponding structure is given in eq. S2 below.

$$\Delta\varphi = n_{eff}\frac{2\pi}{\lambda}\Delta d \qquad (S2)$$

For instance, differences in phase accumulations were determined as 21.74π and -32.6π radian along the PhC slab region with the length of 4.4μm at 0.26 ($\omega$a/2π) and 0.32 ($\omega$a/2π), respectively (Fig. S2). Therefore, at such frequencies different effective indices (1.68 and -2.05 at 0.26 and 0.32 ($\omega$a/2π), respectively) were obtained. Even though the calculated field profiles may involve the various reflections and refractions in the structure, it is important to observe the change in the phase evolution for two different frequencies.

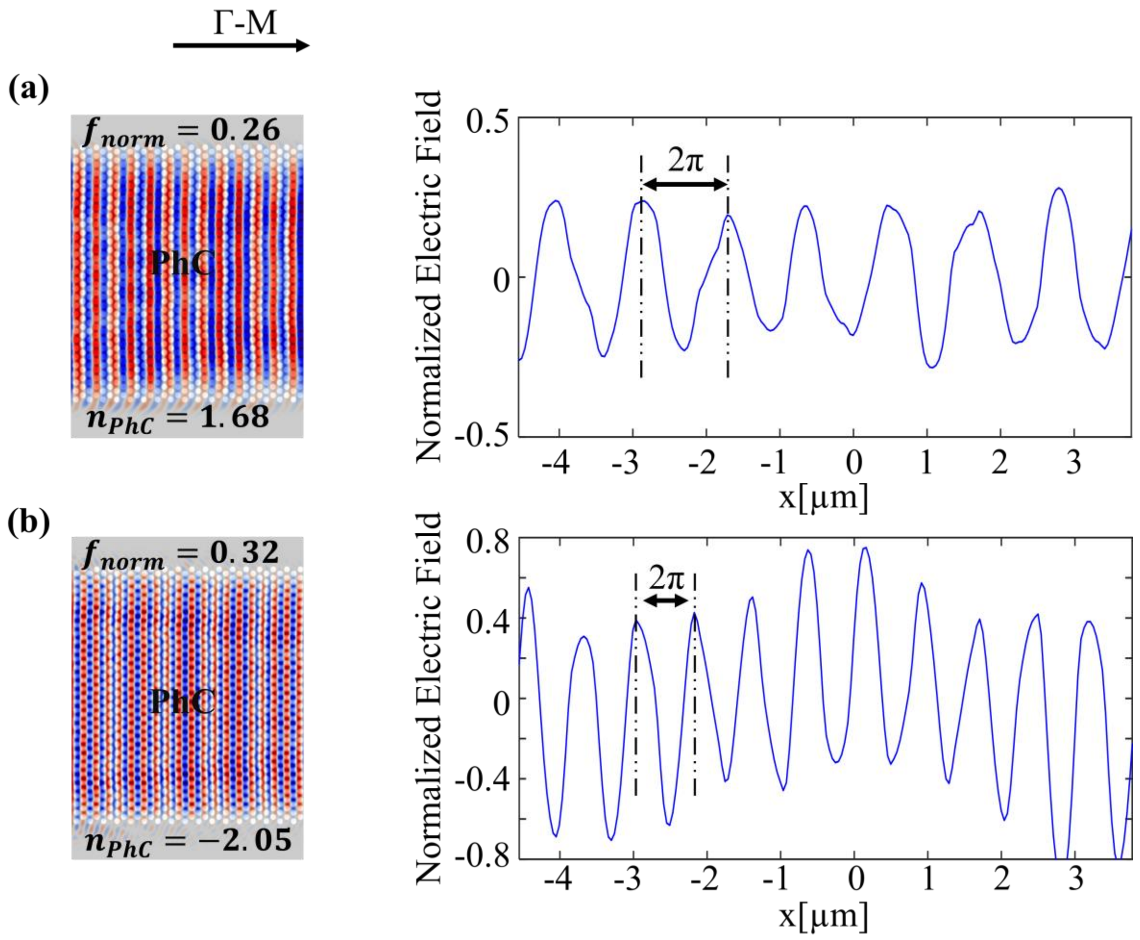


**Figure S2.** Electric field distributions (left) and amplitude profiles of the electric field along optic axis (Γ-M) (right) for the structure of an air-hole slab based hexagonal lattice with $h = 0.6a$ and $r = 0.3a$ where $a = 0.5\mu m$ at **(a)** 0.26 ($\omega$a/2πc) and **(b)** at 0.32 ($\omega$a/2πc).

## III. Plasma Dispersion Effect

The change of refractive index for most materials could be created by applying electric field. Conventional methods such as Pockel, Kerr and Franz-Keldysh effects could not provide enough index change in silicon bulk due to the lattice structure of silicon. On the other hand, plasma dispersion effect, which is the effect of carriers injected inside the material on the change in refractive index, is suitable for telecommunication purposes.

The relation between the injected carrier density and refractive index change in silicon was demonstrated experimentally by Soref and Bennet [46] in 1987. Based on the results of these experiments, the

refractive index changes due to electron and hole carrier injection can be found with eq. S3 and eq. S4 given below.

$$\Delta n_e = -8.8 \times 10^{-22} \times (\Delta N_e) \qquad (S3)$$

$$\Delta n_h = -8.5 \times 10^{-18} \times (\Delta N_h)^{0.8} \qquad (S4)$$

In the equations, $\Delta N_e$ and $\Delta N_h$ are the electron and hole carrier concentrations in the silicon, respectively. The phase shifter used in the proposed modulator is composed of silicon PhC slab. In order to obtain index change of 0.01, the effects of both electron and hole carriers was used. A p-i-n diode structure was designed and after applying required voltage, it was proposed that both electron and hole carriers with density of $4.1 \times 10^{18}$ cm$^{-3}$ were accumulated in the intrinsic region of the diode. Such carrier densities provided the required index change in silicon according to the eq. S3 and eq. S4. The refractive index change obtained with different concentrations of carriers is shown in Fig. S3.

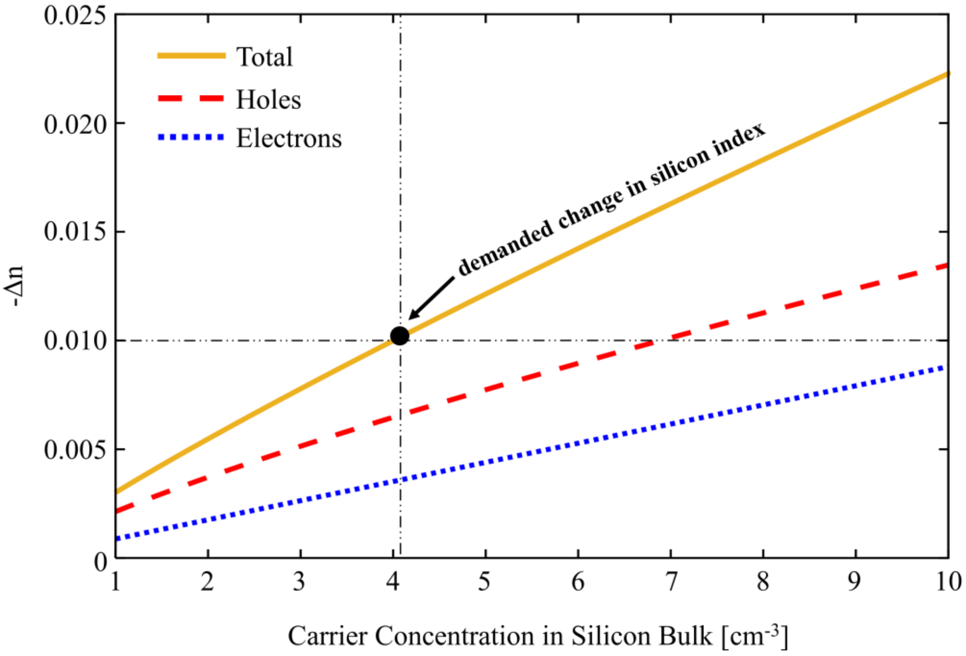


**Figure S3.** The effect of carrier in silicon bulk on the refractive index change

# IV. P-I-N Diode Analysis

## a. Determining P-I-N Diode Parameters

The p-i-n diode structure used to accumulate free carrier in PhC region was specified via solutions of the Poisson and electron-hole continuity equations which are given below.

$$\nabla\varepsilon \cdot \nabla\emptyset = -q(p - n + N_D - N_A) \quad \text{(S5)}$$

$$\frac{dn}{dt} = \frac{1}{q}\nabla.\left(q\mu_n nE + qD_n\frac{dn}{dx}\right) \quad \text{(S6)}$$

$$\frac{dp}{dt} = -\frac{1}{q}\nabla.\left(q\mu_p pE - qD_p\frac{dp}{dx}\right) \quad \text{(S7)}$$

In Poisson equation (eq. S5), ε, $\emptyset$, $p$, $n$, $N_D$ and $N_A$ represent the permittivity of the material, electrostatic potential, hole concentration, electron concentration, donor concentration and acceptor concentration, respectively. In continuity equations (eq. S6) and (eq. S7), the change of the carrier concentrations over time is governed by drift and diffusion mechanisms. The first terms in the parenthesis are the representations of the drift component where $\mu_n$ and $\mu_p$ are the electron and hole mobilities and $E$ is the electric field which is the reason for the formation of the drift mechanism. The second terms are the presentments of diffusion component which shows the transportation of the carriers when concentration gradient exists. In diffusion terms $D_n$ and $D_p$ stand for the diffusivity of the electron and hole particles. By inserting $E = -\nabla\emptyset$ relation to the continuity equations, we actually have three partial differential equations depending on $p$, $n$ and $\emptyset$ parameters. The continuity equations coupled to Poisson equation were solved iteratively via Sentaurus device simulator. Moreover, the effects of different recombination mechanisms such as radiative, Auger and Shockley-Read-Hall (SRH) recombination were considered. Radiative, Auger and SRH recombination rate equations are as follows:

$$R_{radiative} = A\left(np - n_i^2\right) \quad \text{(S8)}$$

$$R_{Auger} = \left(B_n n + B_p p\right)\left(np - n_i^2\right) \quad \text{(S9)}$$

$$R_{SRH} = \frac{np - n_i^2}{\tau_p(n + n_1) + \tau_n(p + p_1)} \quad \text{(S10)}$$

In these equations $A$, $B_n$ are $B_p$ are radiative and Auger rate coefficients and $n_i$ is the intrinsic carrier concentration all which depend on the material selection. SRH recombination rate defines the material quality through lifetime and trap energy level. In eq. S10, $\tau_n$ and $\tau_p$ represent the electron and hole lifetimes and they were taken equal in the simulations. Also, $n_1$ and $p_1$ were assumed to be equal to $n_i$ meaning that the trap energy level is located at the midpoint of the energy band gap.

## b. Capacitance and Resistance of the Diode

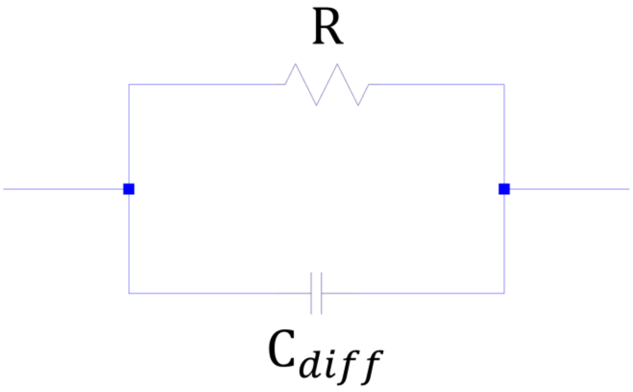


**Figure S4.** Schematic representation for the electrical model of the p-i-n diode

The electrical model of the p-i-n diode for forward bias action is shown in Fig. S4. For the forward biasing, the capacitance is due to the diffusion of minority carriers from p and n doped regions to the intrinsic region. The behavior of electron and hole carriers was examined during the determination of diode characteristics. The total charge in the intrinsic region can be obtained from carrier concentration in that region for different biasing voltages by using the eq. S11 given below.

$$Q = q \times (n + h) \times \mathrm{V} \qquad \text{(S11)}$$

where $q$, $n$, and $h$ are the electron charge ($1.6 \times 10^{-19}$ C), electron and hole carrier concentrations, respectively. V represents the volume of the intrinsic region, which is $\mathrm{V} = 1.91\mu m \times 5.788\mu m \times 0.286\mu m = 3.16\mu m^3$. Electron and hole carrier concentrations are the same in the proposed diode structure. The total charge in the intrinsic region for different biasing voltages is shown in Fig. S5a. The diffusion capacitance can be obtained with the relation (eq. S12) between total charge and voltage.

$$C = \frac{dQ}{dV}\bigg|_{V_{operation}} \qquad \text{(S12)}$$

$$R = \left(\frac{dI}{dV}\right)^{-1}\bigg|_{V_{operation}} \qquad \text{(S13)}$$

The capacitance changes with the voltage applied to the contacts. For different voltages the capacitance is shown in Fig. S5b. At the DC operation point for the proposed design, which is 1.091 V, the capacitance is 24 pF.

The resistance of the diode can be determined from the current-voltage plot (Fig. S5c). The inverse slope of the plot gives the resistance (eq. S13). The p-i-n diode resistance is 125 Ω.

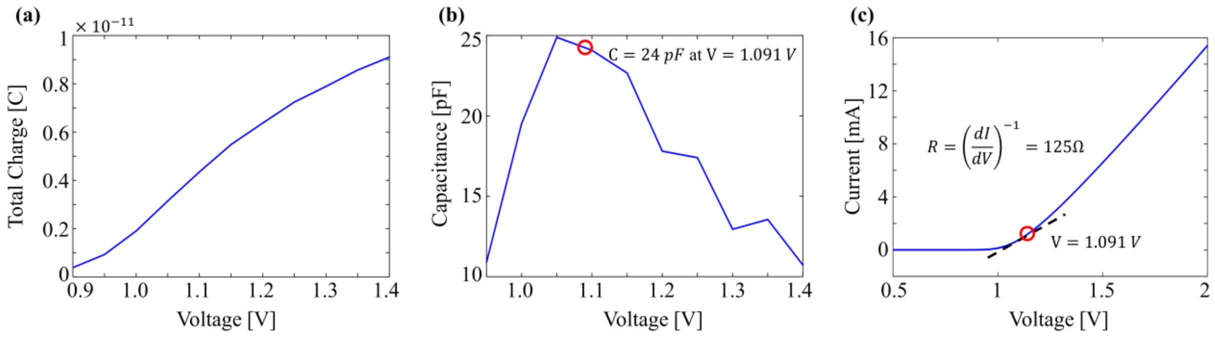


**Figure S5.** Total charge in the intrinsic region **(a)**, diffusion capacitance of the p-i-n diode at forward bias **(b)**, and current passing through the diode **(c)** for different voltage

## V. Insertion Losses in the Structure

The insertion loss in the arms of the proposed MZI has been determined from field transmission results by FDTD simulations. Calculated insertion losses including the reflections at the interfaces are ~1.08 dB and ~1.05 dB for off-state and on-state of the modulator, respectively. The losses were calculated from the transmission of the reference arm (no p-i-n diode) and device arm (with biased p-i-n diode) of the MZI during on-state ($V_{DC} = 1.091V$). The corresponding transmission plot is shown in Fig. S6.

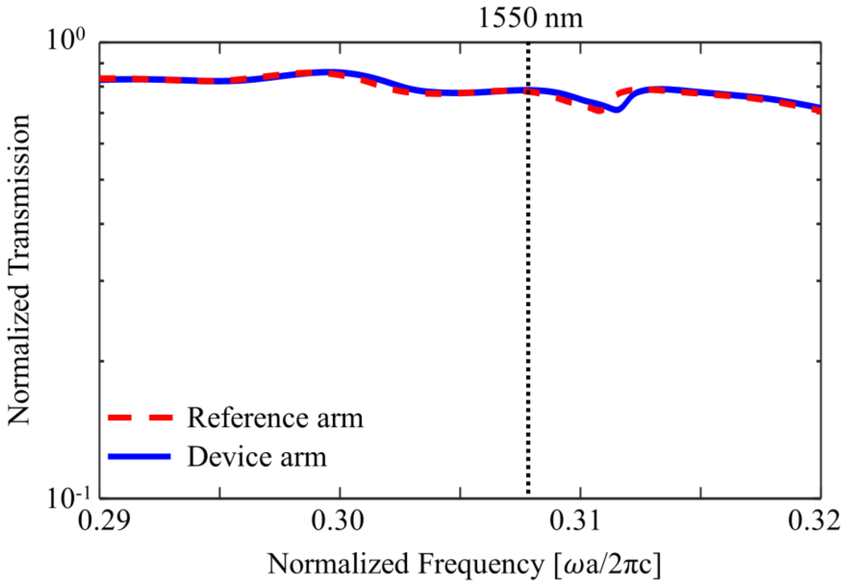


**Fig. S6.** Optical transmission spectrum along the two arms of the MZI at on-state ($V_{DC} = 1.091V$)
